# Observations on the Preservation of Sight, and on the Use, Abuse, and Choice of Spectacles, &c.

**Published:** 1834-04-01

**Authors:** 


*Observations on the Preservation of Sight, and on the Use, Abuse, and Choice of Spectacles, Reading-Glasses, &c.* By John Harrison Curtis, Esq. London, 1834. Small 8vo. pp. 48.

The design of this treatise is good; not so the execution. First of all, there is far too little in the book; and then Mr. Curtis is continually falling into a most disagreeable error of Hygienic writers, who are fond of representing the most innocent things as dangerous and destructive; who would wish to make us eat, drink, and sleep in Sanctorius's chair, like the hypochondriac in the Spectator; or would advise us to lie down on the rug after dinner, like a desperate Abernethian. Thus, we are told, at p. 11, that "rubbing the eyes on waking is a destructive habit, which many people have contracted." A destructive habit! Pshaw! Our author wants to frighten his dark-eyed readers, and tells them that their eyes "are weaker, and more susceptible of injury from various causes, than grey or blue eyes." (P. 23.) We would recommend the dark-eyed to console themselves with the reflection, that in those countries where the solar light is most intense eyes are generally black. Mr. Curtis says, "The lighter the pupil, the greater and longer-continued is the degree of tension the eye can sustain." (P. 23.) By *pupil* the author means the *iris:* but what does *tension* mean? Exertion?

When short-sighted people want glasses, Mr. Curtis says, "I would strenuously advise all such to be satisfied with glasses as slightly concave as possible: by which I mean, that they should employ no higher power than is necessary to enable them to see distinctly objects at from forty to fifty feet distance." (P. 43.) Objects of what size? Very great, or very little? Beer's Augenkrankheiten, or the Observations on the Preservation of the Sight? The truth is, that, to try the power of distant vision, objects of well-known size should be selected as the standard: for instance, a page printed in small pica may be placed before the doubting student; and, if he cannot read it with facility at six inches distance, he is short-sighted.

Before taking leave of Mr. Curtis, we cannot but suggest to him, that the referring us for testimonials of the value of his work on the Eye to the Literary Gazette, Court Journal, Town, Beau Monde, &c. has the air of a wicked jest, and should be omitted in his future advertisements.

